# Optimal diagnosing and interventional treatment of the posterior ligamentous complex inflammatory syndrome

**DOI:** 10.1016/j.inpm.2025.100609

**Published:** 2025-07-03

**Authors:** Bunty Shah, Yakov Vorobeychik

**Affiliations:** Department of Anesthesiology and Perioperative Medicine, Penn State M.S. Hershey Medical Center, 500 University Dr. Hershey, PA, 17033, USA

## Abstract

**Introduction:**

The previously described posterior ligamentous complex inflammatory syndrome can result in chronic axial low back pain. This condition can be identified through MRI findings that demonstrate inflammatory changes in the compartments of the posterior ligamentous complex region, with the space of Okada serving as a connection between them. However, an effective interventional treatment for this syndrome has not yet been proposed.

**Case:**

We present the case of a patient suffering from persistent axial low back pain who did not respond to medication or physical therapy. A SPECT scan revealed significant radiotracer uptake in the bilateral L4-L5 facet joints and the L4-L5 interspinous ligament. Given that bilateral L3-L4 diagnostic medial branch blocks yielded negative results, posterior ligamentous complex inflammatory syndrome was suspected. Injection of contrast dye into the L4-L5 interspinous adventitial bursa demonstrated the spread of contrast material from the injection site to the space of Okada and the bilateral L4-L5 facet joints. Subsequent steroid injection provided the patient with over 80 % pain relief at the five-week follow-up.

**Conclusion:**

Patients experiencing axial low back pain, particularly those with negative diagnostic medial branch blocks, should consider undergoing a SPECT scan. This recommendation is particularly relevant in cases involving Baastrup disease or pars defects, as these conditions are often associated with the presence of the space of Okada, which is crucial for the development of PLCIS. If this diagnosis is confirmed through imaging, a steroid injection into the adventitial interspinous bursa may offer an effective treatment for PLCIS by facilitating medication distribution throughout the compartments of the posterior ligamentous complex region.

## 1 Introduction

In their radiological study, Lehman et al. reported lumbar MRI findings indicative of inflammatory changes in various posterior structures of the lumbar spine, including bilateral facet joints, adventitial interspinous bursa, and the retrodural space of Okada [1]. The authors posited that these observed inflammatory changes might be associated with low back pain, coining the term "posterior ligamentous complex inflammatory syndrome (PLCIS)" to aptly characterize these findings. Drawing upon their personal clinical experiences, they noted that axial back pain predominates in the clinical presentation of PLCIS; however, they refrained from recommending specific therapeutic interventions for this condition. They underscored the pivotal role of the space of Okada in developing PLCIS, emphasizing its function as a conduit that interconnects multiple compartments involved in this inflammatory process.

The space of Okada was first identified in the cervical spine by Kikuzo Okada in 1981 during an anatomical study of cervical facet joints [2]. The study revealed a communicating pathway located dorsal to the ligamentum flavum in 80 % of the 142 joints examined. This pathway connects the facet joint to the interlaminar and interspinous regions, the contralateral facet joint, and the cervical extradural space. This retrodural space is confined to the interlaminar area and is not continuous in the coronal plane. It is rarely observed in the lumbar spine, except in instances of pars interarticularis defects or Baastrup disease (kissing spine) [1,[3], [4], [5]]. The prevalence of PLCIS remains undetermined; however, studies investigating contrast flow during CT- and fluoroscopic-guided injections in the lumbar region have reported instances of aberrant contrast flow in the space of Okada, with rates ranging from 0.6 % to 7.5 % [[6], [7], [8], [9], [10]].

We present a very unusual case involving a patient with persistent chronic axial low back pain who did not respond to conservative treatment. The patient was diagnosed with PLCIS by a CT-SPECT scan and was successfully treated with a steroid injection into the interspinous adventitial bursa.

## 2 Case

A patient presented to the interventional pain medicine clinic with a complaint of axial low back pain that has persisted for several years. The patient characterized the pain as predominantly aching, rating its intensity at 10 out of 10 on the numeric rating scale. Previous conservative treatment approaches, including nonsteroidal anti-inflammatory drugs, muscle relaxants, and physical therapy, have proven ineffective in alleviating the pain.

**Fig. 1:**
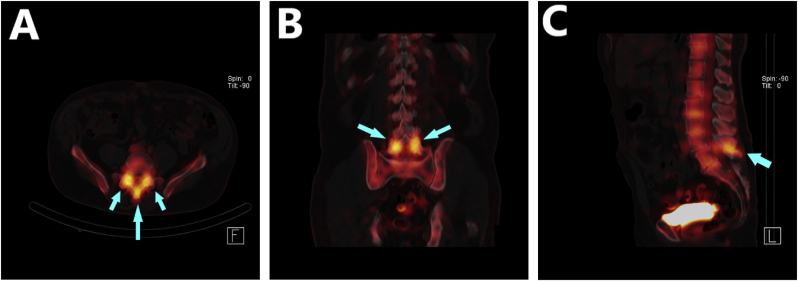
NM Bone Three-Phase Study w**ith SPECT.** Axial **(A)** and coronal **(B)** views reveal intensive radiotracer uptake within the bilateral L4-L5 facet joints. Sagittal view **(C)** indicates pronounced radiotracer activity in the L4-L5 interspinous bursa. Basstrup disease, commonly referred to as “kissing spine,” is evident at this anatomic level. Light blue arrows indicate areas of increased radiotracer uptake. (For interpretation of the references to colour in this figure legend, the reader is referred to the Web version of this article.)

The physical examination revealed no significant findings, except for increased discomfort during bilateral extension-rotation of the lumbar spine and localized tenderness to palpation in the midline and slightly bilaterally along the paraspinal region at the L4-L5 vertebral level. Lumbar spine radiographs obtained prior to his visit showed moderate to severe multilevel degenerative disc changes and grade 1 anterolisthesis of L4 on L5. Subsequent radiographs taken in flexion and extension ruled out any dynamic instability related to the noted anterolisthesis. A Single-Photon Emission Computed Tomography (SPECT) scan, ordered for further evaluation, revealed intense radiotracer activity in the bilateral L4-L5 facet joints and the L4-L5 interspinous ligament associated with L4-L5 Baastrup disease (Fig. 1). A diagnostic medial branch block targeting the bilateral L3 and L4 medial branches of the dorsal rami was performed using 0.5 ml of 2 % Lidocaine per nerve; however, this intervention did not reduce pain scores post-injection.

Based on the results of the SPECT scan and negative diagnostic medial branch block, we established the diagnosis of PLCIS and decided to perform a steroid injection into the L4-5 adventitial interspinous bursa. A formal informed consent for this procedure was duly obtained.

**Fig. 2:**
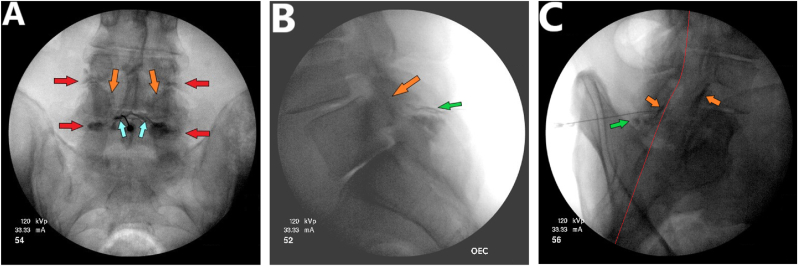
Contrast flow pattern in the posterior ligamentous complex region after injection of Iohexol 240 into the L4-L5 adventitial interspinous bursa. **(A)** Anterior-posterior view shows a collection of contrast medium in the space of spreading in both L4-L5 facet joints and extending into the superior and inferior capsular recesses. **(B)** Lateral view illustrates linear dye spread within the L4-L5 interspinous bursa and arthrogram of the L4-L5 facet joints. **(C)** Oblique view shows the filling of the L4-5 facet joints with contrast and the presence of a small collection of contrast in the space of Okada. The distribution of the contrast material dorsal to the ventral interlaminar line (VILL) indicates the extradural location of the needle. *(Light blue arrows: space of Okada; Red arrows: superior and inferior capsular recesses; orange arrows: facet joints, green arrows: advential interspinous bursa; red line: ventral interlaminar line (VILL).). (For interpretation of the references to colour in this figure legend, the reader is referred to the Web version of this article.)*

Following the placement of a 22-gauge, 3.5-inch spinal needle in the L4-L5 interspinous bursa, 3 ml of Iohexol 240 was injected. Conventional anterior-posterior and lateral fluoroscopic views demonstrated contrast flow in all compartments of the posterior ligamentous complex region, including the L4-L5 adventitial interspinous bursa, the space of Okada, and the bilateral L4-L5 facet joints (Fig. 2A and B). The oblique view (Fig. 2C) confirmed the extradural placement of the needle, positioned dorsal to the ventral interlaminar line (VILL) [11,12].

**Fig. 3:**
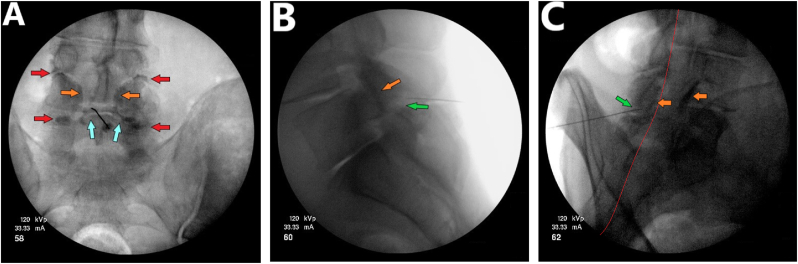
Contrast flow pattern following injection of the therapeutic solution. **(A)** Anterior-posterior view shows a notable attenuation of the contrast medium in the space of Okada, alongside marked opacification of the bilateral L4-L5 facet joints. **(B)** Lateral view demonstrates that the previously observed contrast within the L4-L5 interspinous bursa has been dispersed (“washed out”), while a significant accumulation of contrast material in the L4-L5 facet joints is evident. **(C)** Oblique view indicates the presence of contrast in the space of Okada and its accumulation in the L4-L5 facet joints. *(Light blue arrows: space of Okada; Red arrows: superior and inferior capsular recesses; orange arrows: facet joints, green arrows: advential interspinous bursa; red line: ventral interlaminar line (VILL).). (For interpretation of the references to colour in this figure legend, the reader is referred to the Web version of this article.)*

Subsequent injection of 40 mg of triamcinolone diluted in 1 ml of 1 % Lidocaine resulted in the clearance of contrast material from the L4-L5 interspinous bursa, alongside the collection of dye in the space of Okada and marked opacification of both L4-L5 facet joints (Fig. 3). At the 5-week follow-up after the injection, the patient reported more than 80 % pain relief and significant improvement in his functional status.

## 3 Discussion

Despite extensive efforts to identify the source of pain, the specific pain generator remains undetermined in a significant number of patients suffering from axial chronic back pain. In some instances, these patients may have PLCIS as the underlying cause of their pain. Although MRI may be diagnostic in identifying PLCIS as the source of pain, it is contraindicated in patients with implanted pacemakers, defibrillators, intracranial aneurysm clips, cochlear implants, and various other conditions involving metallic objects or implants within the body. Additionally, numerous insurance providers routinely deny coverage for this study in patients presenting with axial low back pain in the absence of accompanying neurological symptoms, which may result in many individuals going undiagnosed. A SPECT scan represents a more cost-effective alternative that is typically more readily approved by insurance companies. Furthermore, this imaging modality may demonstrate non-inferiority or even superiority to MRI in diagnosing PLCIS as the source of low back pain.

Interestingly, in the presented case, the diagnostic medial branch block did not provide any post-injection pain relief, despite significant inflammatory activity observed in the L4-5 facet joints on the SPECT scan. While it is understood that both lumbar facet joints and the interspinous ligament are primarily supplied by the medial branches of the lumbar dorsal rami [13], the absence of pain relief following the diagnostic medial branch block in this case suggests the possibility that nociceptive signals originating from the posterior ligamentous complex may be transmitted through nerves other than, or in addition to, the medial branches. Conducting further anatomical studies to identify the sources of innervation for the lumbar interspinous ligament could provide valuable insights into this case.

Injection of medication into any compartment of the posterior ligamentous complex region should result in its distribution to the other compartments through the space of Okada. Several case reports documented the unintentional flow of contrast within the space of Okada during various injection procedures, including facet joint injections [14] as well as interlaminar [[6], [7], [8], [9],[15], [16], [17]] and transforaminal ESI [[18], [19], [20]]. However, achieving needle entry into degenerated facet joints is not consistently feasible, and there is no reliable way to access the space of Okada, which is typically entered accidently during procedures [21]. In fact, the dye flow patterns observed in the space of Okada are consistently regarded as aberrant [22]. Consequently, placing the needle within the adventitial interspinous bursa is the most straightforward and reliable approach to delivering therapeutic solutions into all posterior ligamentous complex region compartments.

## 4 Conclusion

Patients experiencing axial low back pain, particularly those with negative diagnostic medial branch blocks, should consider undergoing a SPECT scan. This recommendation is particularly relevant in cases involving Baastrup disease or pars defects, as these conditions are often associated with the presence of the space of Okada, which is crucial for the development of PLCIS. If this diagnosis is confirmed through imaging, a steroid injection into the adventitial interspinous bursa may offer an effective treatment for PLCIS by facilitating medication distribution throughout the compartments of the posterior ligamentous complex region.

## Declaration of competing interest

The authors declare that they have no known competing financial interests or personal relationships that could have appeared to influence the work reported in this paper.
